# Hearing training with mobile application for tinnitus: usability and clinical results of the Zumit app

**DOI:** 10.1590/2317-1782/e20250005en

**Published:** 2026-02-09

**Authors:** Izabella Lima de Matos, Rubens Jonatha dos Santos Ferreira, Maria Fernanda Capoani Garcia Mondelli

**Affiliations:** 1 Programa de Pós-graduação em Fonoaudiologia, Faculdade de Odontologia de Bauru – FOB, Universidade de São Paulo – USP - Bauru (SP), Brasil.

**Keywords:** Tinnitus, Mobile Applications, Auditory Training, Usability Testing, Personal Satisfaction

## Abstract

**Purpose:**

To evaluate usability and clinical impact of an application for tinnitus identification and intervention.

**Methods:**

This was a pre- and post-test observational study. A convenience sample included individuals with tinnitus complaints and normal hearing who were on a waiting list at a teaching clinic. Audiological assessment included conventional and high-frequency audiometry, immittance testing, tinnitus pitch matching, Tinnitus Handicap Inventory, and Visual Analog Scale. The intervention consisted of auditory training. The application was assessed for efficiency, effectiveness, and participant satisfaction using the System Usability Scale and Net Promoter Score. The Shapiro-Wilk test assessed normality. Paired t-tests and Wilcoxon tests were applied, adopting a 5% significance level. Missing data were excluded from final analysis.

**Results:**

From the sample of 29 participants, 23 completed the final evaluation. 47.8% were male and 52.2% female. Mean age of 46 years. All had experienced tinnitus for over six months. 39% reported a hissing sound, 43.5% a whistling sound, and 17.5% a cicada-like sound. Usability assessment showed an average score of 77.06, with 50% of participants classified as promoters, 25% as neutral, and 25% as detractors. Pre- and post-intervention results with the Zumit app demonstrated a statistically significant reduction in Visual Analog Scale scores (W = -231.000, p < 0.001) and a decrease of 21.478 points (p < 0.001) in the overall Tinnitus Handicap Inventory score.

**Conclusion:**

The Zumit app featuring frequency discrimination auditory training, may be an effective tool for tinnitus management. Limitations include the sample size and restricted generalization to individuals with normal audiometry.

## INTRODUCTION

Tinnitus is the perception of a non-existent sound in the environment, which may develop into a disorder when it causes emotional distress, cognitive and physical difficulties, leading to behavioral changes and daily life limitations^(1)^. Its prevalence is approximately 14% among the global adult population, with 2% experiencing severe tinnitus^(2)^.

Authors^(3)^ have identified significant complaints related to tinnitus, such as emotional disturbances, social interaction difficulties, work-related issues, and general health dysfunctions, with prevalence rates between 7% and 19% among individuals with tinnitus. This condition is regarded as a public health concern, with increasing demand for identification and effective treatment^(4)^. However, tinnitus evaluation and intervention remain challenging for various healthcare professionals.

Although tinnitus is an auditory symptom, consensus on its characterization and measurement is lacking^(5)^. Literature^(6)^ indicates that psychoacoustic measures are essential to obtain relevant diagnostic information about the symptom's subjective intensity and severity. These parameters are crucial for guiding and selecting appropriate treatment, quantifying its effects, and providing tangible evidence of tinnitus perception reduction. Beyond diagnostic complexity, tinnitus therapy is also challenging due to its multifactorial nature. One of the main challenges in tinnitus management lies in bridging the gap between diagnostic complexity and the need for effective interventions. Among therapeutic alternatives, auditory training (AT) stands out for its positive outcomes in tinnitus patients^(7)^.

AT involves active stimulation of the auditory nervous system, aiming to develop new auditory skills. By being exposed to specific and systematic sound stimuli, listeners enhance their ability to differentiate sounds, and the auditory system reorganizes in response to these demands, demonstrating neural plasticity^(8)^. Authors^(7,8)^ report that tinnitus is associated with deficits in binaural integration and selective attention, cognitive processes critical for effective auditory perception, making AT a viable therapeutic option for these patients. The literature^(7)^ also indicates that patients exposed to daily auditory training sessions show significant benefits in at least one outcome measure of the studies.

Nevertheless, access to AT remains limited due to the distribution of professionals and resources within the healthcare system.

A potential solution to this scenario is the use of technology-mediated healthcare as a facilitator and decentralizer of intervention tools. Recently, there has been growing interest in developing smartphone applications to assist in tinnitus management and treatment^(9,10)^. Offering a validated application for tinnitus identification and therapy aims to address the needs of individuals whose quality of life is severely affected by the condition. Therefore, investments in alternatives that support the development and implementation of evaluation and treatment strategies for individuals in healthcare services are justified. This study aims to evaluate the usability (ease of use, accessibility, and adherence) and clinical impact (symptom reduction and functional improvement) of a mobile application developed for tinnitus identification and intervention.

## METHOD

This study is characterized as an observational, pre- and post-test design. The study was conducted following approval from the Research Ethics Committee of a higher education institution under opinion no. 51088521.9.0000.5417 and with the consent of patients for voluntary participation and data publication, confirmed through the signing of the Free and Informed Consent Form. The study was conducted between November 2022 and June 2023.

To include the largest possible number of users, the Zumit application was developed for two platforms: Android (version 5 or higher) and iOS (version 9 or higher), providing core functionalities aimed at achieving the objectives of this study. The app development process was detailed in the study by Lima et al.^(11)^, following the Contextualized Instructional Design method, which involves planning/analysis, development, and practical application, with new tools incorporated as needed. The app offers a range of features, including: detailed patient registration, application of the Tinnitus Handicap Inventory (THI) questionnaire to assess the impact of tinnitus on quality of life, graphical visualization of tinnitus discomfort levels via the Visual Analog Scale (VAS), generation of personalized auditory stimuli (pure tones and narrowband noise), detailed user activity logs, direct communication with healthcare professionals, interactive gamification features to enhance patient engagement, and tools for monitoring and managing treatment by professionals.

Auditory training was conducted through frequency discrimination activities, performed by participants over the period previously established by the speech-language pathologist researcher. In the application, individuals listen to the presented auditory stimuli and select the icon corresponding to the question: "Which sound is lower or higher in pitch?" Additionally, users can observe the remaining time and the number of correct and incorrect answers, followed by a demonstration of the auditory stimulus. In the case of an incorrect response, patients are encouraged to attempt again. The sounds available in the application for auditory training include frequencies of 125, 250, 500, 750, 1k, 1.5k, 2k, 3k, 4k, 6k, 8k, 9k, 10k, 11.2k, 12.5k, 14k, 16k, 18k, and 20k Hz. For each frequency, two types of continuous sounds were selected: pure tone and narrow-band noise, both presented at maximum intensity (level 1) with a duration of three seconds^(11)^.

Patient adherence to the auditory training activities, the number of unique achievements completed, the current training day, and the date of the last session were monitored through app-generated statistics linked to the researcher's management profile. Additionally, the application features a "Chat with the Patient" option, which the researcher used to address participants' questions regarding their therapeutic process, further supporting treatment adherence^(11)^.

The sample was delineated by considering the population with tinnitus who seek care at the audiology teaching clinic of Faculdade de Odontologia de Bauru da Universidade de São Paulo. This facility is accredited by the Sistema Único de Saúde – SUS and provides public services, registering 40 and 25 new weekly cases, respectively, totaling 20,000 patients per year diagnosed with hearing loss and fitted with hearing amplification devices at the affiliated university hospital, and 5,000 patients per year at the audiology clinic. Among the new weekly cases, approximately 20% do not meet the criteria established for care within the hearing health service—that is, they do not have a diagnosis of hearing loss as defined by ordinances GM 793 of April 24, 2012, and GM 835 of April 25, 2012—yet they seek the service for tinnitus treatment following medical referral.

Accordingly, studies were initiated to support this population that does not require amplification and, as a result, cannot benefit from the use of individual sound generators. The study in question provides these individuals with a treatment at low cost to the public health system and under professional supervision. Therefore, the development of the study follows a methodology that considers the need for health services to address the gap in care for this specific population, using a convenience sample based on the demand of individuals referred by physicians and the fulfillment of the participant selection criteria established for this study.

To ensure reliable results, adults with continuous tinnitus and normal hearing were selected, excluding those taking ototoxic medications or undergoing other tinnitus treatments. The selection of patients with tinnitus and normal hearing was justified by the need to reduce the influence of hearing loss on the auditory skills required for auditory training, given that this was the initial test of the application. Further studies involving populations with different auditory profiles are currently being conducted to investigate the effectiveness of this application.

All participants underwent a comprehensive audiological evaluation, and those meeting the inclusion criteria (continuous tinnitus complaints, age 18 years or older, normal hearing) were granted access to the Zumit app. Individuals with cognitive or motor impairments, those who did not consent to participate, and those without a compatible smartphone were excluded.

Participants underwent audiological assessments, including conventional tonal audiometry, classified according to the World Health Organization^(12)^, and extended high-frequency evaluation from 8 kHz onward, specifically at 9, 10, 11.2, 12.5, 14, 16, and 20 kHz. Evaluations were conducted in a controlled acoustic environment, inside an acoustically treated booth, using the AC40 audiometer (Interacoustics) and HH200 headphones, both properly calibrated and up to date. High-frequency audiometry identified potential hearing losses at higher frequencies, while acoustic immittance measurements helped detect middle and inner ear alterations. The normality values defined by Klagenberg et al.^(13)^ were considered.

Tinnitus characterization was conducted through tinnitus matching, an assessment that determines the perceived sound's frequency and intensity. Participants also completed the THI and VAS scales to evaluate the impact of tinnitus on quality of life and the level of discomfort, respectively. These instruments were applied before and after the intervention to enable comparisons over time.

Participants used the Zumit app for personalized auditory training aimed at reducing tinnitus impact. After registration and identifying tinnitus characteristics via the THI and VAS questionnaires, participants began a six-week auditory training program. The app provided personalized auditory stimuli tailored to each participant's needs. Treatment monitoring was conducted remotely, with healthcare professionals available to address questions and provide guidance. After six weeks of intervention, participants were reassessed using the same THI and VAS questionnaires applied at the study's onset.

The Zumit app was evaluated for efficiency, effectiveness, and participant satisfaction. Items analyzed included the visibility of app elements, language use, task-related information, and the likelihood of recommending the app to others. To assess user interaction with the app, the System Usability Scale (SUS) usability test was applied. The usability questionnaire was adapted from the original model^(14)^, as in another study^(9)^ on app development for the tinnitus population.

The Net Promoter Score (NPS) was also used, guided by the question: "On a scale of zero to ten (0 to 10), how likely are you to recommend this app to someone?" Responses were categorized as follows: Promoters (scores of 9 or 10), considered satisfied users who encourage others to use the app; Neutrals (scores of 7 or 8), who neither promote nor deter app use; and Detractors (scores of 0 to 6), indicating dissatisfaction. The overall satisfaction score was calculated by subtracting the percentage of detractors from the percentage of promoters. Scores of 75-100% were considered excellent, 50-74% very good, 0-49% fair, and -1 to -100% poor^(15)^.

Data collected during the study were transferred to a Microsoft Excel spreadsheet and analyzed using descriptive qualitative statistics, with inferential analysis applied. The Shapiro-Wilk test was used for normality analysis, and paired t-tests and Wilcoxon tests were applied, with a 5% significance level adopted. Missing data and participant losses during the study were handled by excluding those cases from the final analysis. Participants were monitored through the application's built-in tracking system, which recorded their engagement with the proposed activities. Completion of these activities was the primary criterion for inclusion in the final analysis; therefore, individuals who did not complete the follow-up or failed to perform the required tasks as monitored by the application were excluded from the final dataset.

## RESULTS

A total of 116 individuals enrolled in the study, but only 29 were included in the sample as they met all established criteria. Of these 29 participants, 23 completed the initial evaluation, finished the auditory training (AT), and participated in the final evaluation. The dropout rate in this study was 20.7%, resulting from the failure to complete the planned activities and/or the participant's absence in the final evaluation. Thus, the results of the Zumit application evaluation were assessed using two tools: the SUS questionnaire and NPS.

Among the 23 participants considered for the study results, 47.8% were male and 52.2% were female. The average age of the participants was 46 years. Regarding tinnitus duration, all participants had experienced tinnitus for more than six months. In terms of sound characterization, 39% described their tinnitus as a hissing sound, 43.5% as a whistling sound, and 17.5% as a cicada-like sound.

The overall average SUS score was 77.06 (57–78; SD = 8.30), indicating no major usability issues and categorizing the score as very good. The NPS scale showed that 50% of the evaluators were classified as promoters, meaning they were satisfied and encouraged others to use the app. Meanwhile, 25% were neutral, not actively promoting the app but not discouraging its use either; and 25% were detractors, indicating dissatisfaction. This highlights good user satisfaction with the Zumit app as measured by the NPS scale.

To compare the overall THI scores before and after the intervention, the data passed the Shapiro-Wilk normality test (P = 0.052). Thus, a paired t-test was used to compare pre- and post-intervention THI total scores. Table 1 demonstrates a reduction of 21.47 points in the overall average score before and after the intervention with the app.

**Table 1:** Comparison of overall THI questionnaire scores before and after the intervention

**Condition**	**N**	**Mean**	**Standard deviation**
Overall THI - Before	23	44.783	18.759
Overall THI - After	23	23.304	16.729
**Difference**	**23**	**21.478**	**11.973**

**Source:** Study authors

**Caption:** t = 8.603 (P ≤ 0.001)

In comparing the Functional domain of the THI questionnaire before and after the intervention, the analysis failed the Shapiro-Wilk normality test (P < 0.050). Consequently, the Wilcoxon test was applied for the pre- and post-condition comparison. Table 2 shows a six-point difference in the overall median, suggesting a reduction in scores before and after the intervention.

**Table 2:** Comparison of the THI questionnaire Functional domain before and after the intervention

**Condition**	**N**	**Median**	**25%**	**75%**
Functional THI - Before	23	14.000	10.000	24.000
Functional THI - After	23	8.000	4.000	14.000

**Source:** Study authors

**Caption:** W = -213.000 (P ≤ 0.001)

For the Emotional domain of the THI questionnaire, the data passed the Shapiro-Wilk normality test (P = 0.102). Therefore, a paired t-test was used for pre- and post-condition comparison. Table 3 shows an 8.5-point reduction in the Emotional domain scores before and after the intervention with the app.

**Table 3:** Comparison of the THI questionnaire Emotional domain before and after the intervention

**Condition**	**N**	**Mean**	**Standard deviation**
Emotional THI - Before	23	15.913	8.728
Emotional THI - After	23	7.391	6.366
Difference	23	8.522	6.097

**Caption:** t = 6,703 (P = <0.001)

In the Catastrophic domain of the THI questionnaire, the analysis passed the Shapiro-Wilk normality test (P = 0.148). Consequently, a paired t-test was conducted to compare conditions. Table 4 highlights a 4.5-point reduction in scores before and after the intervention with the app.

**Table 4:** Comparison of the THI questionnaire Catastrophic domain before and after the intervention

**Condition**	**N**	**Mean**	**Standard deviation**
Catastrophic THI - Before	23	11.478	3.871
Catastrophic THI - After	23	6.957	5.218
Difference	23	4.522	3.964

**Caption:** t = 5,470 (P = <0.001).

Finally, in comparing the Visual Analog Scale (VAS) scores before and after the intervention, the analysis failed the Shapiro-Wilk normality test (P < 0.050). Thus, the Wilcoxon test was used for pre- and post-condition comparison. Similar to the THI results, there was a reduction in the median VAS scores before and after the use of the app, as shown in Table 5. A statistically significant difference was observed (p < 0.001) in the total score, indicating a substantial reduction in discomfort. The initial median score was 8.00, which decreased to 5.00 by the end of the study, representing a three-point reduction.

**Table 5:** Comparison of VAS scores before and after the intervention

**Condition**	**N**	**Median**	**25%**	**75%**
VAS - Before	23	8.000	5.000	9.000
VAS - After	23	5.000	3.000	7.000

**Caption:** W= -231,000 (P = <0.001)

## DISCUSSION

The present study developed and evaluated a mobile application aimed at supporting both professionals and individuals suffering from tinnitus, presenting itself as a tool that may assist in providing low-cost intervention options. In addition to developing a mobile application with features useful for both patients and professionals, it is important to assess its usability.

In this regard, authors^(9)^ emphasize that such an evaluation is crucial to verify the use of a product and investigate aspects involving user navigation and interface comprehension. Therefore, it is necessary to understand the types of tinnitus-focused applications available in app stores, as well as scientific studies on their features, to analyze their usability and reliability.

The overall usability score of the Zumit app was 77.06, which is considered satisfactory. A similar result was observed in another usability study^(16)^, which also used the SUS questionnaire to evaluate the usability of the Dizziness Kids App. In that study, approximately 80% of the participants disagreed with needing assistance from others to use the app. A theoretical usability study using SUS^(14)^ suggests that, based on the parameters evaluated in the questionnaire, an appropriate score ranges from 70 to 100. Scores below 70 indicate that the product may present serious usability issues.

Another evaluation metric used for the Zumit app was the NPS scale, which showed high user satisfaction, with 50% of evaluators being promoters—satisfied users who encourage others to use the app. A similar finding was observed in a study(9) evaluating the AVAZUM app, developed to assist in the initial assessment of tinnitus, with an NPS score of 58%.

The NPS scale is widely used to measure customer satisfaction with products, services, and other offerings. The score is determined by the percentage of promoters exceeding the percentage of detractors. This indicates that the product is more likely to be recommended by its users. Neutral or passive users are not dissatisfied but are unlikely to recommend it to others. Authors^(17)^ suggest that an NPS score of 50% or higher indicates very good satisfaction, while scores below 49% suggest insufficient satisfaction.

Using these usability evaluation metrics in innovative health technology products is essential, as this validation ensures the tool meets the minimum requirements for user comprehension and ease of use.

Regarding clinical findings for tinnitus, a reduction was observed in all THI subscales and the VAS scores. Assessing the impact of the symptom is extremely important as it aids in guiding the individual and understanding their actual situation, influencing engagement in the proposed intervention. Authors^(4)^ suggest that the THI is a key instrument as it is widely used in clinical routines, enabling an analysis of the discomfort caused by tinnitus and the evaluation of intervention benefits. Like the THI questionnaire, the VAS also proves to be an important tool as it allows point-specific evaluation and monitoring of the intervention, supported by visual aids. Its use is becoming common as a quantitative tool offering values to measure the degree and annoyance of tinnitus reported by patients^(18)^.

To date, no consensus exists regarding intervention in tinnitus cases, posing challenges for professionals in managing the symptom. Sound therapy is a strategy being explored for these cases, involving the incorporation of auditory stimulation into the individual’s daily life to alleviate the discomfort caused by tinnitus. An international study^(19)^ aimed to review types of sound therapy and analyze how different characteristics of tinnitus patients influence their therapeutic effects, providing a reference for personalized tinnitus sound therapy selection. At the end of the study, the authors noted that personalized sound therapy includes auditory discrimination training, which can effectively suppress tinnitus in some patients, but they emphasize the need for further research on the subject.

The intervention method determined in the present study was AT with frequency discrimination. Initially used to improve auditory processing skills, this strategy is being investigated as an ally in tinnitus management, with promising results ranging from reduced attention to the symptom to improvements in its perceived intensity^(20)^.

Authors^(21)^ estimated the effects of AT in reducing tinnitus distress and improving auditory skills in elderly individuals with hearing loss using hearing aids. They observed statistically significant differences in THI scores and behavioral tests before and after treatment. These results corroborate the findings of the present study, as statistically significant differences were observed in the total THI score and all assessed domains.

The positive results observed in this study for both the THI and VAS questionnaires, comparing pre- and post-intervention with AT, align with recent findings^(22)^ evaluating the effects of AT on tinnitus perception using subjective questionnaires. The study found that the perceived severity and disadvantage of tinnitus decreased following frequency discrimination-based treatment.

The dropout rate observed in the study (20.7%) may be associated with various factors, including challenges related to the app's usability, barriers to participant engagement, and individual differences in tinnitus severity. Firstly, although the average usability score of the Zumit app was considered satisfactory (77.06 on the System Usability Scale), interface or workflow difficulties may still exist, discouraging participants from continuing. Additionally, engagement in digital interventions depends on factors such as adequate technical support, ease of access, and continuous motivation, which may have varied among users. Another possible explanation is the heterogeneity in tinnitus perception: individuals with milder symptoms may not feel the need to complete the treatment, whereas those with more severe tinnitus may have unmet short-term expectations, leading to frustration and dropout.

Although the results are positive, it is important to note that this study analyzed only individuals with normal hearing. This factor contributed to the small sample size. The limitations of this study should also be considered, including the absence of complementary objective measures, the lack of qualitative data collection, the short-term usability assessment (six weeks), which does not account for long-term adherence and sustained benefits, the lack of a control group as a methodological limitation, and the need for a clearer exploration of the generalizability of the results to different population profiles. Therefore, it is crucial to conduct further studies using the Zumit app and frequency discrimination-based AT as interventions in larger populations with varying audiological conditions to provide broader insights into its potential applicability.

## CONCLUSION

The usability results demonstrate that Zumit is an efficient and effective app that fosters user satisfaction and recommendation. Tinnitus patients who used AT for six weeks through the Zumit app showed significant improvement in participation restriction, reflected in both the total score and the three THI domains. The benefits were also confirmed by significant changes in VAS scores before and after the intervention. Thus, it can be suggested that the Zumit app, combined with frequency discrimination-based AT, can be an allied tool in tinnitus management. The findings should be interpreted with caution, considering that the sample consisted solely of individuals with normal hearing and that adherence was limited. Nevertheless, it is important to conduct further studies involving its use and evaluation in individuals with tinnitus associated with different audiological conditions.
